# Preparation and Characterization of Iron-Doped Tricalcium Silicate-Based Bone Cement as a Bone Repair Material

**DOI:** 10.3390/ma13173670

**Published:** 2020-08-19

**Authors:** Yanan Zhang, Jiapan Luan, Yin Zhang, Shuai Sha, Sha Li, Shanqi Xu, Dongqing Xu

**Affiliations:** 1 College of Materials Science and Engineering, Nanjing Tech University, Nanjing 211816, China; zyn3648@njtech.edu.cn (Y.Z.); 201861103064@njtech.edu.cn (J.L.); 201961203152@njtech.edu.cn (S.S.); 201961103082@njtech.edu.cn (S.L.); 201961103075@njtech.edu.cn (S.X.); 4201170305@njtech.edu.cn (D.X.); 2 Nanjing Haoqi Advanced Materials Co., Ltd., Nanjing 211300, China

**Keywords:** bone cement, tricalcium silicate, Fe-doped, biocompatibility

## Abstract

Iron is one of the trace elements required by human body, and its deficiency can lead to abnormal bone metabolism. In this study, the effect of iron ions on the properties of tricalcium silicate bone cement (Fe/C_3_Ss) was investigated. It effectively solved the problems of high pH value and low biological activity of calcium silicate bone cement. The mechanical properties, in vitro mineralization ability and biocompatibility of the materials were systematically characterized. The results indicate that tricalcium silicate bone cement containing 5 mol% iron displayed good self-setting ability, mechanical properties and biodegradation performance in vitro. Compared with pure calcium silicate bone cement (C_3_Ss), Fe/C_3_Ss showed lower pH value (8.80) and higher porosity (45%), which was suitable for subsequent cell growth. Immersion test in vitro also confirmed its good ability to induce hydroxyapatite formation. Furthermore, cell culture experiments performed with Fe/C_3_Ss ion extracts clearly stated that the material had excellent cell proliferation abilities compared to C_3_Ss and low toxicity. The findings reveal that iron-doped tricalcium silicate bone cement is a promising bioactive material in bone repair applications.

## 1. Introduction

Bone tissue defects and injuries are common diseases in clinic. Bone defect sites, usually caused by aging, trauma and other reasons, do not have the ability to complete self-repair, which usually seriously affects people’s normal life [1,2]. De Grado et al. and Winkler et al. reviewed the existing shortcomings, clinical applications and future prospects of various bone repair materials for treating bone defects [3,4]. At present, among many methods to treat bone defects, traditional bone repair techniques are still used in clinic, such as autogenous bone, allogeneic bone, metal materials, etc. [5]. In addition to autogenous bone with limited bone mass, other biomaterials do not yet possess good biocompatibility, degradability and porous three-dimensional structure. Due to the inherent shortcomings of these materials, traditional bone repair techniques are limited in curing bone defect diseases [6].

To overcome the deficiency of traditional bone repair materials, modern bone repair materials have been rapidly developed, and bone cement is one of them. Some studied have found that bone cement biomaterials, such as calcium phosphate, calcium silicate and calcium sulfate, have good biocompatibility, self-setting behavior and can be shaped arbitrarily [7,8,9]. For example, Ho et al. found calcium silicate bone cement had good bioactivity and osteogenic ability, and it could be used as bone defect repair material [10]. Bone cements have excellent prospects for bone repair, but there are also many deficiencies, such as limited mechanical strength and biological activity. At the same time, better degradation rates, suitable setting time and porosity and mineralization capabilities are also problems that need to be further studied and solved in bone cement materials [11,12,13]. In addition, enhancing the osteogenic ability of bone cement materials to accelerate bone healing can improve the repair effect of bone defects, which is of great significance in clinic.

Among bone cement biomaterials, calcium silicate-based materials have received extensive attention due to their excellent osteogenicity, biodegradability and mechanical strength [14,15,16,17]. Calcium silicate can also degrade in vivo and release silicon element for promoting human bone growth [18]. Tricalcium silicate (C_3_S) is a typical calcium silicate-based biomaterial, which has a major role in the field of orthopedics due to its self-setting property, biological activity and the ability to fill irregular shape defects [19]. Although C_3_S has many advantages, the biological activity is still low and needs to be further improved. At the same time, the hydration of C_3_S generates the highly basic byproduct calcium hydroxide (Ca(OH)_2_), which is rapidly ionized and modified to generate Ca ions and OH^−^ [20]. Ca ion can promote osteogenic differentiation and mineralization of bone cells [21,22], while free OH^−^ may increase pH value and result in inflammation [19]. Iron, an important basic element in bone tissue, plays a key role in bone growth and metabolism [23]. The formation of Ca(OH)_2_ can be reduced by incorporating Fe^3+^ into the C_3_S compound to replace a part of Ca^2+^. Furthermore, the ion release and metabolism of iron-containing biomaterials have also attracted researchers’ attention [24]. Zhang et al. reported that mouse bone marrow stromal cells cultured on Fe-doped tricalcium phosphate had good adhesion morphology and proliferation capacity, and the expression of osteogenesis-related genes was significantly enhanced [25]. Xia et al. also confirmed that the introduction of iron into calcium phosphate cement scaffolds can enhance bone regeneration [26]. Therefore, introducing iron into bone cement may be beneficial to improve the cell proliferation capability.

In this study, a new type of iron-containing biological bone cement was prepared; the influences of different concentrations on C_3_S bone cement properties, such as setting time, compressive strength, porosity and anti-washout property were investigated; and in vitro experiments were systematically performed. An in vitro immersion study was conducted in simulated body fluids (SBF). The mineralization ability of Fe-doped tricalcium silicate bone cement (Fe/C_3_S) biomaterials was evaluated by XRD, FT-IR and SEM. Moreover, mouse fibroblasts (L929 cells) were utilized to evaluate the cell proliferation ability and cytotoxicity of Fe/C_3_Ss bone cement materials.

## 2. Materials and Methods

### 2.1. Preparation of Pure C_3_S and Fe-Doped C_3_S Powders

C_3_S and Fe-doped tricalcium silicate (Fe/C_3_S) powders were synthesized by sol–gel method [27]. Briefly, 2-M nitric acid solution (HNO_3_, as a catalyst), absolute ethanol (C_2_H_5_OH, as a solvent), calcium nitrate tetrahydrate (Ca(NO_3_)_2_·4H_2_O, XiLong Chemical Co., Ltd., Shantou, China) and ferric nitrate nonahydrate (Fe(NO_3_)_3_·9H_2_O, Saan Chemical Technology Co., Ltd., Shanghai, China) were sequentially added to tetraethyl orthosilicate (TEOS, Sinopharm group Co., Ltd., Shanghai, China). Then, it was stirred well for 1 h. The molar ratio of TEOS-HNO_3_-C_2_H_5_OH was 10:10:1. The iron ion accounted for 0, 2.5, 5 and 10 mol% of the total metal cation concentration, respectively. Then, the above mixed solution was aged at 60 °C for 48 h, followed by drying for 48 h at 120 °C. Next, the gel was heated to 1450 °C at a rate of 5 °C/min, and then kept in a high temperature resistance furnace for 8 h. Finally, the sintered powder was finely ground in an agate mortar and sieved with a 300 mesh for further studies.

### 2.2. Preparation of Bone Cement

The C_3_S or Fe/C_3_S powder was mixed with 0.5-M dipotassium hydrogen phosphate solution, which served as curing liquid. The liquid to powder ratio was 0.3 mL/g. When stirred into paste, the solution was promptly filled into the mold (Φ 6 mm × 3 mm or Φ 6 mm × 12 mm) to obtain a unified shape for the following experiments. The Fe/C_3_S bone cements were named as Fe-0, Fe-2.5, Fe-5 and Fe-10 according to the Fe-doped concentrations of 0, 2.5, 5 and 10 mol%, respectively.

### 2.3. Material Properties Evaluation

According to ASTM C266-15 [8], the final setting time of samples (Φ 6 mm × 12 mm, n = 4) was measured by utilizing the Gilmore needle indentation technique. Setting time was determined by Gilmore needle (1 mm in diameter and 453 g in weight). The setting time was recorded from when the solid–liquid phase was uniformly mixed to when the Gilmore needle could not produce a 1-mm deep indentation on the surface of mixture.

The preparation of C_3_Ss and Fe/C_3_Ss samples (Φ 6 mm × 12 mm, n = 4) were carried out by the above method. The demolded samples were placed in a curing box at 37 °C and 100% relative humidity for 72 h. Thereafter, those samples were dried at 37 °C for 24 h and polished to measure the compressive strength. The compressive strength of bone cement sample was tested through an electronic universal testing machine (TFW-2S, Tuofeng Instrument Co., Ltd., Shanghai, China), and the cross head descending rate was 0.5 mm/min.

According to ISO standard 39231/1-1979(E) [28], the porosity of C_3_Ss and Fe/C_3_Ss samples was measured by liquid displacement (Archimedes) method. Firstly, the samples (Φ 6 mm × 12 mm, n = 4) were dried and then weighed to get dry weight. Secondly, the samples were suspended in deionized water to obtain suspended weight. Finally, the samples were completely immersed in deionized water and vacuum filtered to record wet weight. Equation (1) was used to calculate the porosity value of the sample.

(1) $$Porosity(\%)=\frac{(M_{3}-M_{1})\times \rho }{M_{3}-M_{2}}\times 100\%$$

where *ρ*, *M*_1_, *M*_2_ and *M*_3_ are, respectively, the density of water, dry weight, suspended weight and wet weight of the samples.

### 2.4. In-Vitro Immersion Study

Experiments can be performed in human simulated body fluids (SBFs), which are used for in vitro immersion [29]. The preparation of SBF was based on a previous study [30]. The samples (Φ 6 mm × 3 mm) of C_3_Ss and Fe/C_3_Ss were immersed in SBF solution at 37 °C for 1 h. The samples’ surface morphology before and after immersion were observed and compared. If there is no obvious particle collapse or dispersion, it indicates that bone cement has a good anti-washout property [31].

The pH values of each bone cement sample (Φ 6 mm × 3 mm, n = 4) soaked in SBF solution for different times (0, 1, 3, 5 and 7 days) were detected with a pH meter (PHSJ-3F, Yidian Instrument Co., Ltd., Shanghai, China) [32]. The liquid–solid ratio was 20 mL/g [33].

The weight loss was used as a function of immersing time to evaluate the biodegradability of the discs (Φ 6 mm × 3 mm, n = 4). The SBF solution was renewed every two days with a solution volume to disc surface area of 10 mL/cm^2^ [34]. After discs were immersed in SBF solution for different time (0, 7, 14, 21 and 28 days), they were dried at 60 °C for 24 h and then weighed. According to Equation (2), the weight loss of the sample was measured.

(2) $$Weightloss(\%)=\frac{M_{5}-M_{4}}{M_{4}}\times 100\%$$

where *M*_4_ and *M*_5_ represent the initial weight of the disc and the dry weight of the disc after immersing different time.

### 2.5. Phase Composition and Microstructure

The phase composition of C_3_S and Fe/C_3_S powders before and after immersion in SBF solution was analyzed by diffraction of X-rays (XRD, Geigerflex, Rigaku, Tokyo, Japan). Fourier transform infrared spectroscopy (FT-IR, NEXUS 670, Nicolet, Beijing, China) further determined the phase composition and chemical bonds of these powders. Surface morphology of discs immersed in SBF solution for 7 days was observed using scanning electron microscopy (SEM, JSM-IT300, JEOL, Tokyo, Japan).

### 2.6. In Vitro Cell Culture Test

The cell viability of L929 mouse fibroblasts (Shanghai TongWei Biotechnology Co., Ltd., Shanghai, China) was used for cell culture test to assess cell proliferation and cytotoxicity [32]. The cells were cultured in the Dulbecco’s modified eagle medium (DMEM) containing 10% fetal bovine serum and stored in incubator with 5% CO_2_ and 95% relative humidity at 37 °C [35]. Before the cell culture experiment, all cement discs (Φ 6 mm × 3 mm) were sterilized at 160 °C for 8 h. The C_3_Ss and Fe/C_3_Ss discs were immersed in DMEM for 3 days at 4 °C to prepare ion extracts, and the solid to liquid ratio was 0.2 g/mL [36]. The cultured cells were planted in the extract for 24, 48 and 72 h, respectively, to evaluate the cell proliferation abilities and toxicity tests of the materials.

#### 2.6.1. Cell Proliferation

The proliferation of L929 cells in four different extracts was investigated by cell counting kit (CCK-8, Beyotime, Shanghai, China). In other words, the cell suspension (10^4^ cells/mL) was separately cultured in 100 μL of extract for 24, 48 and 72 h, respectively. The culture medium and CCK-8 were put in a 96-well plate and maintained in 37 °C incubator for 4 h. After transferring the above mixed solution to a new well plate, the absorbance was read at 450 nm using a micro-plate reader (Bio-Rad 680, Hercules, CA, USA). According to the instruction manual, the cells cultured for 24, 48 and 72 h were qualitatively detected by a live/dead staining kit (Beyotime, Shanghai, China). Cell morphology and distribution were observed by fluorescence microscopy (Zeiss Axioskop 40, Jena, Germany).

#### 2.6.2. Cytotoxicity

Toxicity of the samples to L929 cells was measured by detecting the content of lactate dehydrogenase (LDH, Biyuntian Biotechnology Co., Ltd., Shanghai, China) released into the culture medium. The cells (10^4^ cells/mL) were seeded in ion extracts of four different samples immersed in different periods (24, 48 and 72 h), the supernatant was collected by centrifugation and the absorbance was read using a micro-plate reader to evaluate the LDH activity.

### 2.7. Statistical Analysis

All experimental data were expressed as mean ± standard deviation (SD). Statistical analysis was carried out by student’s test, and the difference was statistically significant (* *p* < 0.05).

## 3. Results

### 3.1. Materials Properties

#### 3.1.1. Setting Time

**Figure 1:**
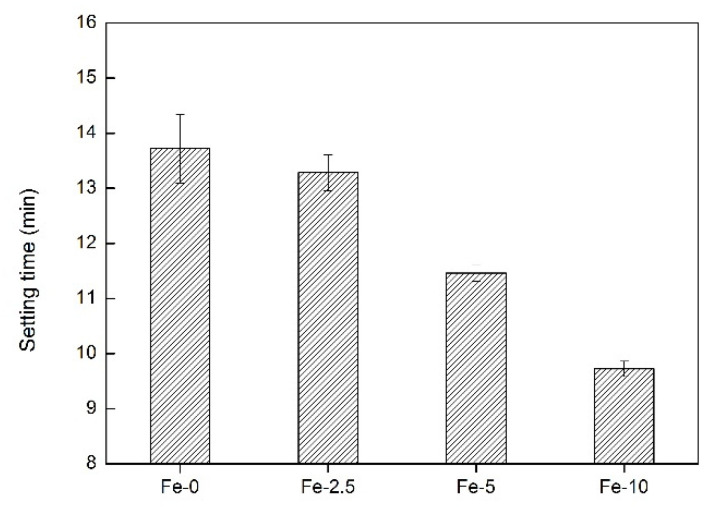
The setting time of Fe-0, Fe-2.5, Fe-5 and Fe-10 bone cement samples.

Setting time is an important indicator of bone cement, which not only provides sufficient operation time for clinical surgery, but also produces curing effect in a short time. The setting time of bone cement should be controlled between 3 and 15 min, which is consistent with the optimal time for surgical operations [37]. Figure 1 shows the setting properties of pure C_3_Ss and Fe/C_3_Ss biomaterials. The results show that from Fe-0 to Fe-10, the setting time of the material is reduced from 13.72 ± 0.57 to 9.72 ± 0.31 min.

#### 3.1.2. Compressive Strength and Porosity

**Figure 2:**
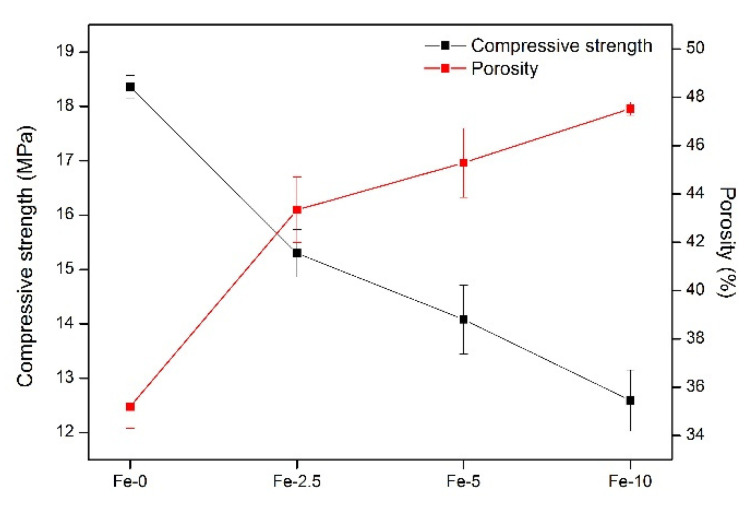
Compressive strength and porosity of Fe-0, Fe-2.5, Fe-5 and Fe-10 bone cement samples.

For orthopedic surgery, it is necessary for bone repair materials to have a certain mechanical strength. Porosity of materials also has a certain influence on the growth of bone cells around the bone defect [38]. Therefore, the compressive strength and porosity of the Fe/C_3_S samples were both measured. As shown in Figure 2, with the increase of iron content, the numerical changes of the compressive strength and porosity of the samples present the opposite trend. For compressive strength, from Fe-0 to Fe-10, the strength value decreases from 18.36 ± 0.21 to 12.59 ± 0.56 MPa. With regard to porosity, the value increases from 35.20 ± 0.48% to 47.53 ± 0.27%.

### 3.2. In-Vitro Immersion Study

#### 3.2.1. The pH Value

**Figure 3:**
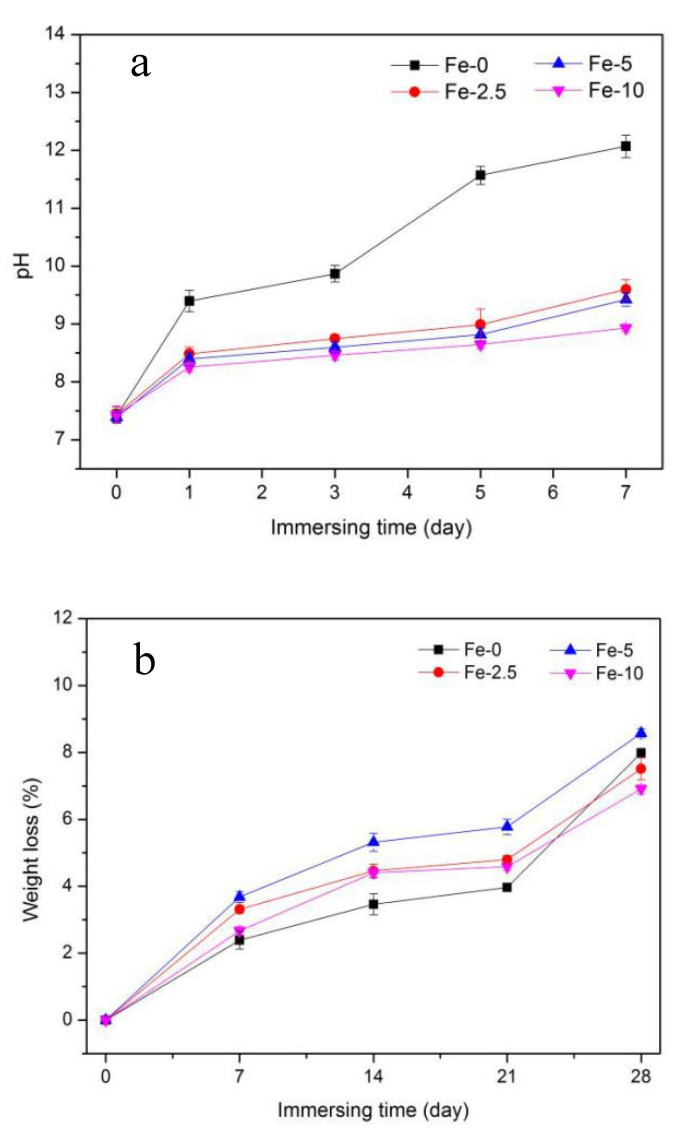
pH value (**a**); and weight loss (**b**) of Fe-0, Fe-2.5, Fe-5 and Fe-10 bone cement samples after immersing in SBF solution.

The pH values of Fe-0, Fe-2.5, Fe-5 and Fe-10 samples are indicated in Figure 3a. The change of pH value is mainly due to the exchange of ions between the SBF solution and the samples. It can be seen that the pH value of the solution after immersion increases with the extension of the immersion time. In addition, after immersing for the same time, the pH value of Fe-0 sample is higher than that of the three other samples. The pH values of Fe/C_3_Ss samples are not significantly different, which can effectively reduce the higher pH value of pure C_3_Ss to a certain extent.

#### 3.2.2. Weight Loss

Figure 3b shows the weight loss of C_3_Ss and Fe/C_3_Ss samples after immersing for different times in SBF solution (0, 7, 14, 21 and 28 days). After immersing for 28 days, the weight loss rate of the materials was greater than other immersing times. For Fe-0, the weight loss rate rose from 2.39 ± 0.26% to 7.99 ± 0.11% during Days 7–28. In the Fe/C_3_Ss samples, the value of Fe-5 increased from 3.68 ± 0.16% to 8.58 ± 0.14%, which was higher than the weight loss of Fe-2.5 and Fe-10. Besides, the degradation rate of the materials after the 21 days of immersion became faster.

#### 3.2.3. Anti-Washout Property

**Figure 4:**
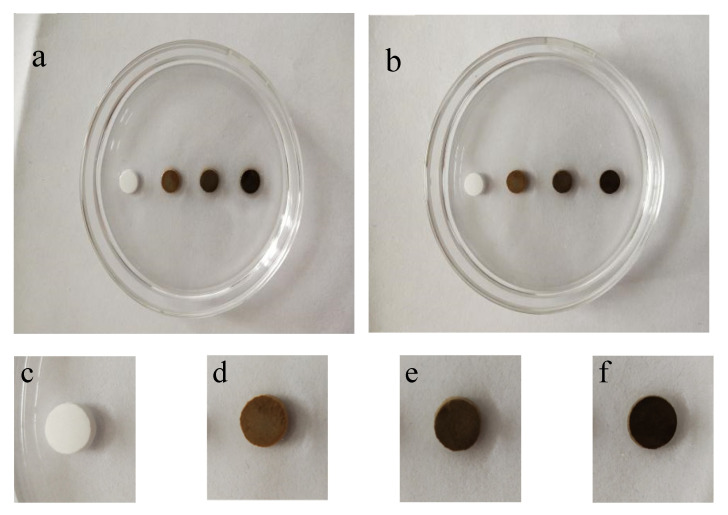
Anti-washout property of Fe-doped cement samples before (**a**) and after (**b**) immersing for 60 min in SBF solution (from left to right are Fe-0, Fe-2.5, Fe-5 and Fe-10); and (**c**–**f**) enlarged pictures of samples after immersing for 60 min, respectively.

Figure 4 displays the anti-washout property of bone cement samples with different Fe contents after immersing in SBF solution for 60 min. As shown in the enlarged images of Figure 4b–f, no particle dispersion phenomenon was observed around the samples, which indicated that the samples have a good anti-washout property.

### 3.3. In-Vitro Mineralization Characterization

**Figure 5:**
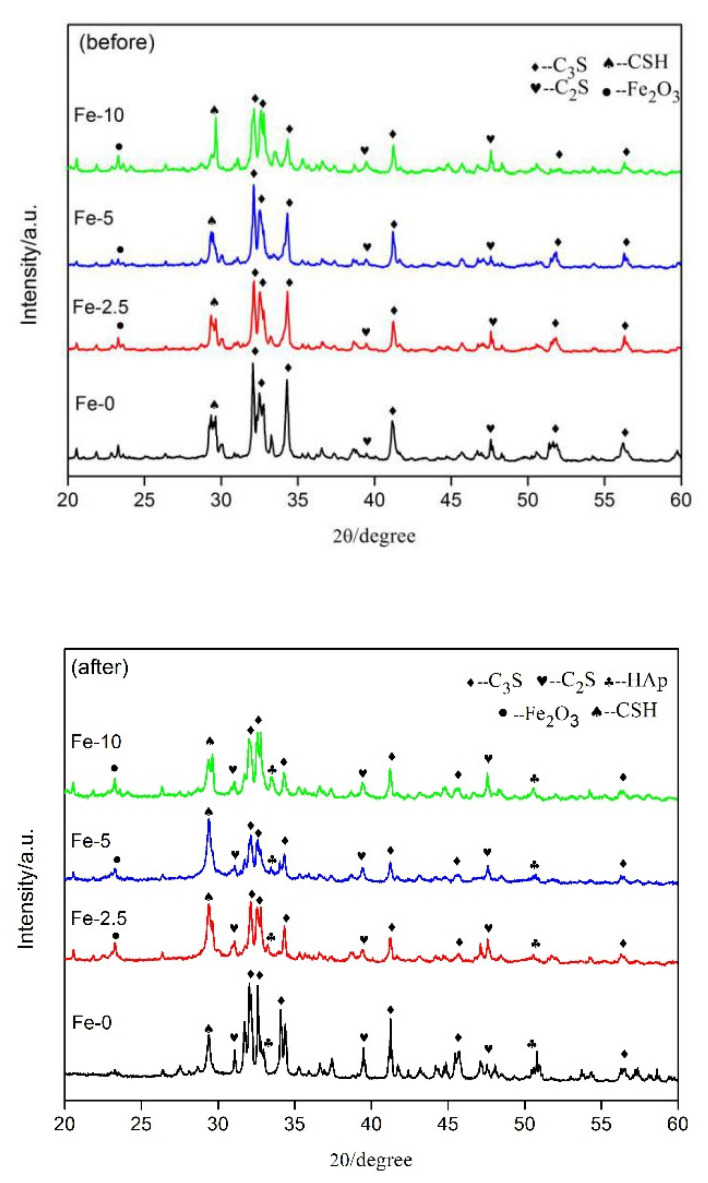
XRD patterns of Fe-0, Fe-2.5, Fe-5 and Fe-10 powders before and after immersing in SBF solution for seven days.

The XRD patterns of C_3_S and Fe/C_3_S powders before and after immersing in SBF solution for seven days are shown in Figure 5. In the pattern before immersing, C_3_S had obvious diffraction peaks at 2θ = 32.1°, 32.5°, 34.3°, 41.1°, 51.7° and 56.3° (JCPDS No. 49-0442), respectively. The diffraction peaks of C_2_S also appeared at 2θ = 32.6°, 41.3° and 47.6° (JCPDS No. 20-0237). In addition, a strong characteristic peak of hydrated calcium silicate (CSH) occurred at 29.4° (JCPDS No. 43-1488), which was the result of hydration of C_3_S or C_2_S. Moreover, the Fe_2_O_3_ phase exhibited a diffraction peak at 23.2° (JCPDS No.39-0238). After immersing, the intensity of the C_3_S diffraction peak decreased somewhat at 32.5°, which may be due to its continued hydration reaction in SBF solution. New diffraction peaks appeared at 2θ = 32.9° and 50.5° (JCPDS No. 09-0432) due to the production of a new phase hydroxyapatite (HAp) after mineralization reaction.

**Figure 6:**
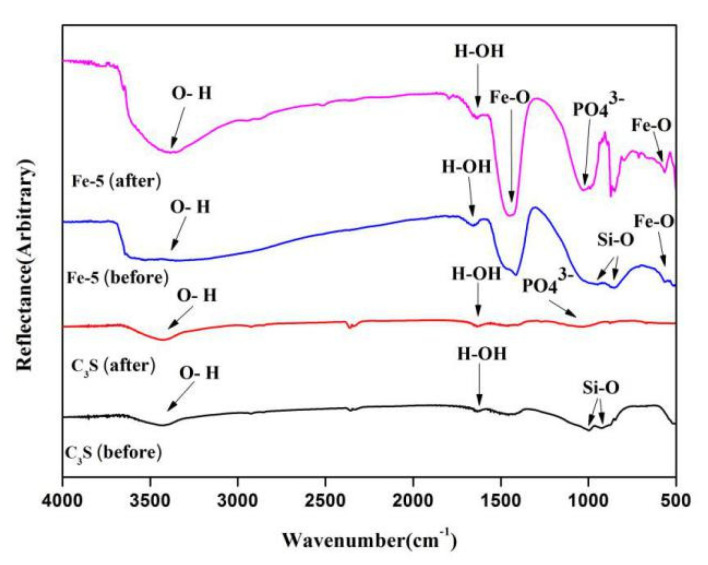
FT-IR spectra of C_3_S and Fe/C_3_S powders before and after immersing in SBF solution for seven days.

Figure 6 shows the FT-IR spectra of C_3_S and Fe-5 powders before and after immersing for seven days in SBF solution. The peaks at 1640 and 3400 cm^−1^ were typical vibrational peaks of H–O–H and O–H bonds of crystalline water in hydrated calcium silicate, respectively [39,40]. The peak at about 1000 cm^−1^ corresponded to the anti-symmetric stretching vibration of the Si–O in C_3_S [40]. After immersing for seven days, a new absorption peak appeared at 1036 cm^−1^ in the FT-IR spectrum. This new peak corresponded to the PO_4_^3−^ asymmetric stretching vibration, which proved the existence of HAp and also confirmed the analysis results of XRD [41]. Besides, the peaks at positions of 561 and 1430 cm^−1^ were the stretching vibration peaks of Fe–O bonds, indicating the presence of Fe_2_O_3_ phase in Fe/C_3_Ss bone cement materials [42].

**Figure 7:**
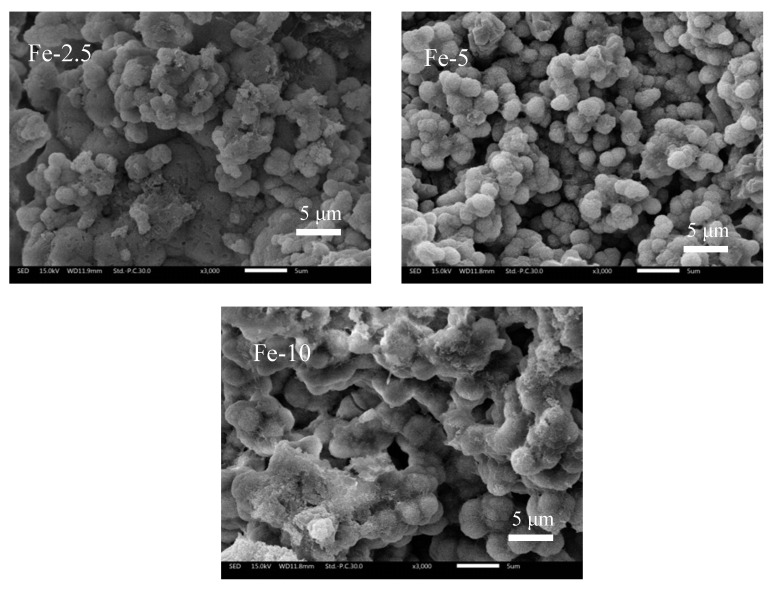
SEM images of Fe/C_3_Ss samples after immersing in SBF solution for seven days.

In Figure 7, the SEM images of the surface of Fe/C_3_Ss samples after immersing in SBF solution for seven days are shown. From the images, it can be seen that the samples were porous and rough. We found many small spherical particles appearing on all sample surfaces (Fe-5 is most obvious). Based on the results of XRD and FT-IR analysis, it could be determined that the spherical particle was HAp, which is similar to our previous study [39]. This shows that Fe/C_3_Ss had certain biological activities in vitro.

### 3.4. Cell Culture

**Figure 8:**
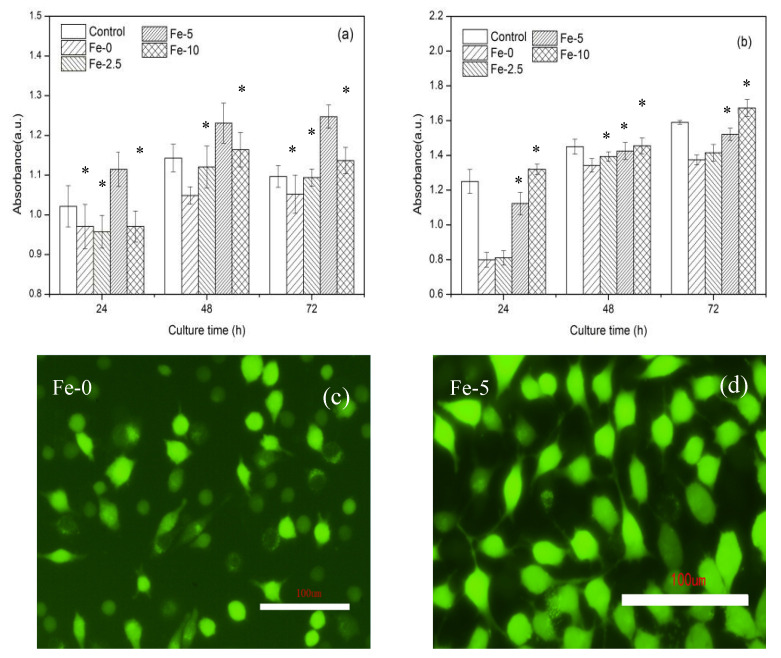
Cell proliferation (**a**) and LDH activity (**b**) after incubation of L929 cells in Fe/C_3_S extract and control (DMEM) for 24, 48 and 72 h and cellular fluorescence pictures (**c**,**d**) after 72 h of incubation in Fe-0 and Fe-5 extracts. Error bars represent means ± SD for n = 4 (* *p* < 0.05).

Figure 8 shows cell proliferation and LDH values after incubation of L929 cells in C_3_Ss and Fe/C_3_Ss extract and control (DMEM) for 24, 48 and 72 h, respectively. Cellular fluorescence pictures after 72 h of incubation in Fe-0 and Fe-5 extract are also exhibited. According to the cell proliferation in Figure 8a, with the extension of culture time, the cell proliferation of Fe/C_3_S samples increased. The cell proliferation of Fe-5 sample was the most obvious and higher than that of the control group and other experiment groups, indicating that Fe-5 could promote cell proliferation.

For the results in Figure 8b, the cytotoxicity also increased slightly. Compared with pure C_3_Ss, Fe/C_3_Ss samples showed a slightly higher cytotoxicity. However, the LDH activity of Fe-5 sample extract was close to that in the DMEM solution., indicating that Fe-5 bone cement material possessed lower toxicity.

Figure 8c,d shows that, when cells were cultured in the extract for 72 h, many living cells (green spots) appeared in the extract of bone cement samples. Compared with pure C_3_Ss, Fe-5 had the largest number of living cells, which is consistent with the results of cell proliferation.

## 4. Discussion

The setting time of the biomaterials required for different operations is different. The factors affecting the setting time of bone cement include temperature, particle size, solid–liquid ratio, powder composition, etc. [43]. On the one hand, iron ions are high-valent cations, which have a compressive effect on the diffused electric double layer of the hydration production CSH colloidal particles; thus, the coagulation of colloidal particles can be accelerated. On the other hand, mainly due to the high porosity of Fe/C_3_Ss samples, when the sample powder was mixed with the curing liquid to form hydrated calcium silicate, it was filled with open pores that were originally filled with water, shortening the setting time [44].

In clinical application, it is necessary for bone cement material to offer adequate mechanical strength support at bone defect sites [37]. Compared with pure C_3_Ss, Fe/C_3_Ss showed lower strength. The main causes were that the valence and radius of calcium and iron ions (0.99 and 0.64 Å) are different and the stable structure of silicon–oxygen tetrahedron was distorted when Ca^2+^ was substituted by Fe^3+^ at its positions and other vacancies [11]. The increase of porosity was related to the certain decrease of compressive strength. After Fe ions replaced more Ca ions, the formation of CSH gel was relatively small. It was difficult to fully fill the gaps between powder particles with Fe content, resulting in increased porosity. At the same time, the addition of nano-oxide could also increase the porosity, which can be observed in SEM images [45].

Owing to C_3_S as the primary component, the hydration reaction occurred in the solution to produce calcium hydroxide (Ca(OH)_2_), a highly alkaline byproduct, which led to the pH value of solution increasing rapidly after immersing for one day. After that, the pH value increased slowly because the formation of hydroxyapatite (HAp) on the samples’ surface partially consumed OH^−^. After immersing for five days, with PO_4_^3−^ depleted, OH^−^ in the solution was no longer consumed. Some unhydrated C_3_Ss continued to be hydrated in SBF solution, which led to the increase of pH value. However, at the same time point, the pH of C_3_Ss was higher than that of Fe/C_3_Ss, which was due to the partial substitution of Ca^2+^ by Fe^3+^ in the preparation of the powders, resulting in a relatively small amount of Ca(OH)_2_.

Biodegradability is one of the significant factors to evaluate the performance of bone cement, and the test process is mainly carried out by means of ion release [1]. Compared with Fe-0 and Fe-2.5, the higher degradation rate of Fe-5 was attributed to its larger porosity and lower strength. It was much easier with larger surface area to degrade in SBF solution. The main reason the weight loss of Fe-10 was lower than that of Fe-5 was that its higher porosity led to its lower density at the same volume and its degradation amount was limited. As the degradation continued, the porosity increased. The increased contact surface between the solution and the material made the material easier to degrade, which explained the acceleration of degradation rate in the later stage of degradation.

Anti-washout performance is the basic clinical requirement for bone cement. The experimental results show that C_3_Ss and Fe/C_3_Ss had a good anti-washout property, which could be explained in two ways. Firstly, the material had large compressive strength and its cohesive force was large. Secondly, it might be related to the formation of calcium silicate gel with high bulk density in SBF solution after immersing.

The SEM images confirmed that the surface of the samples immersed in SBF solution formed a large number of HAp spherical particles. The formation of particles could be explained by the release of Ca^2+^ into the solution and exchange with H^+^ in SBF to form silanol groups (Si–OH). This provided a nucleation site for the formation of HAp. The dense spherical particles on the surface of Fe-5 might be related to the difference in composition and porosity between samples.

In cell experiments, the cell proliferation and fluorescence images well proved that Fe-5 sample had excellent proliferation effect. There are several reasons to explain it. Firstly, the alkalinity of the material has a great influence on proliferation. The closer is the pH value of the material to human body fluid, the better is the cell proliferation. Pure C_3_S has a higher alkalinity, which tends to inhibit cell proliferation and growth. Secondly, the porosity of the sample also has some effects on cell proliferation. In the preparation of extract, Fe-5 had larger porosity than pure C_3_S, which was conducive to the release of Ca and Si ions, and the released ions were beneficial to the osteogenic differentiation of cells, thus promoting cell proliferation. Despite the lower pH value and high porosity of Fe-10, the release of Ca content was limited, which was not conducive to cell proliferation. For Fe-5 with low cytotoxicity, the pH value might be close to that of body fluid, thus it was less harmful to cells. Finally, the introduction of iron ions also had a positive effect on cell culture and bone regeneration [46]. The release level of Si, Ca and Fe ions was a limitation in the current study, which will be considered in the follow-up study. In addition, an animal model study by Matsumoto et al. showed that hydrated calcium silicate cement has a positive interaction with bone tissue [47]. Shi et al. [24] and Ullah et al. [48] also confirmed the positive effects of iron-containing biomaterials on promoting angiogenesis and osteogenic differentiation. Therefore, animal model studies of iron-containing tricalcium silicate bone cement will also be carried out in future studies. Based on the above results, in vitro cell experiments showed that Fe-5 bone cement had good cell compatibility.

## 5. Conclusions

In this study, the iron-containing tricalcium silicate bone cement material was successfully prepared by sol–gel method. Fe/C_3_Ss have some properties that can meet the requirements of clinical surgery, such as better self-curing behavior, excellent biomechanical properties and suitable biodegradation rate. Compared with C_3_Ss, Fe-5 with lower pH value and larger porosity was more suitable for cell growth. At the same time, in vitro mineralization experiments showed that the material had larger degradation rate and good biological activity. In addition, in vitro cell experiments demonstrated that Fe-5 had good cell proliferation and low cytotoxicity, indicating that it had good cell compatibility. Therefore, Fe-doped tricalcium silicate-based bone cement biomaterial is a promising material in the treatment of bone defects.
